# Immune System Dose With Proton Versus Photon Radiotherapy for Treatment of Locally Advanced NSCLC

**DOI:** 10.1016/j.ijpt.2024.100016

**Published:** 2024-04-23

**Authors:** Jimmy S. Patel, Neal S. McCall, Matthew Thomas, Jun Zhou, Kristin A. Higgins, Jeffrey D. Bradley, Sibo Tian, Mark W. McDonald, Aparna H. Kesarwala, William A. Stokes

**Affiliations:** 1 Department of Radiation Oncology, Winship Cancer Institute, Emory University School of Medicine, Atlanta, GA 30322, USA; 2 Department of Radiation Oncology, UPMC Hillman Cancer Center, Pittsburgh, PA 15213, USA; 3 Department of Radiation Oncology, Abramson Cancer Center, University of Pennsylvania, Philadelphia, PA 19104, USA

**Keywords:** Proton, Photon, NSCLC, EDIC

## Abstract

**Purpose:**

Emerging data have illuminated the impact of effective radiation dose to immune cells (EDIC) on outcomes in patients with locally advanced, unresectable non‐small cell lung cancer (NSCLC) treated with intensity-modulated radiotherapy (IMRT). Hypothesizing that intensity-modulated proton therapy (IMPT) may reduce EDIC versus IMRT, we conducted a dosimetric analysis of patients treated at our institution.

**Materials and Methods:**

Data were retrospectively collected for 12 patients with locally advanced, unresectable NSCLC diagnosed between 2019 and 2021 who had physician-approved IMRT and IMPT plans. Data to calculate EDIC from both Jin et al (PMID: 34944813) and Ladbury et al’s (PMID: 31175902) models were abstracted. Paired *t* tests were utilized to compare the difference in mean EDIC between IMPT and IMRT plans.

**Results:**

IMPT decreased EDIC for 11 of 12 patients (91.7%). The mean EDIC per the Jin model was significantly lower with IMPT than IMRT (3.04 GyE vs 4.99 Gy, *P* < .001). Similarly, the mean EDIC per the Ladbury model was significantly lower with IMPT than IMRT (4.50 GyE vs 7.60 Gy, *P* < .002). Modeled 2-year overall survival was significantly longer with IMPT than IMRT (median 71% vs 63%; *P* = .03).

**Conclusion:**

IMPT offers a statistically significant reduction in EDIC compared to IMRT. Given the emergence of EDIC as a modifiable prognostic factor in treatment planning, our dosimetric study highlights a potential role for IMPT to address an unmet need in improving oncologic outcomes in patients with locoregionally advanced NSCLC.

## Introduction

Among lung cancers, approximately 84% are non-small cell lung cancer (NSCLC), with a majority being classified as stage III or IV at diagnosis. Even after the improvements in overall survival (OS) with consolidative durvalumab after chemoradiation, the majority of patients with unresectable or locally advanced NSCLC experience progression within 5 years.^1^, ^2^ Nevertheless, the integration of immune checkpoint inhibitors into the management of NSCLC has generated interest in the interaction between radiation and the immune system. Both preclinical and clinical data indicate that radiotherapy can stimulate an antitumor immune response through immunogenic cell death; however, radiation can also lead to lymphopenia, leading to depletion of the cellular mediators of the immune response and correlating with worse disease outcomes.^3^, ^4^, ^5^, ^6^, ^7^, ^8^, ^9^, ^10^ Radiotherapy therefore constitutes a double-edged sword, potentially promoting tumor control through direct cytotoxic and immunostimulatory effects and compromising tumor response via immunosuppression.

Radiation Therapy Oncology Group (RTOG) 0617, a phase III trial evaluating dose-escalated (74 Gy vs 60 Gy) concurrent chemoradiation therapy with or without cetuximab for patients with unresectable locally advanced NSCLC, provocatively demonstrated that dose escalation adversely impacted both overall survival (OS) and progression-free survival (PFS). Jin et al hypothesized that excess dose delivered to the immune system, independent of cardiopulmonary toxicity, might explain this finding. In a secondary analysis of RTOG 0617, the authors developed a model to calculate the effective radiation dose to immune cells (EDIC) that incorporates radiation dose to the lung and heart, integrative total dose, and number of fractions. In their analysis, higher EDIC correlated not only with lymphopenia but also with worse OS and local control, suggesting the immune system may constitute a novel organ at risk (OAR).^11^, ^12^ Ladbury et al validated these findings in a separate institutional cohort using a model that substituted mean body dose (MBD) for integral total dose (ITD), again identifying an inverse relationship between EDIC and OS.^12^, ^13^

Whether interventions to deliberately reduce EDIC may improve outcomes for patients with locally advanced NSCLC remains unknown. Both the Jin and Ladbury models were initially applied to the plans of patients receiving photon radiotherapy, which deposits dose beyond its intended target and into normal tissues. Due to the innate physical properties of charged particles, proton radiotherapy deposits minimal exit dose in normal tissues, thereby potentially mitigating toxicity and reducing the dose to immune cells.^14^, ^15^, ^16^, ^17^ We hypothesized that proton plans may reduce EDIC as compared to photon plans and therefore conducted a dosimetric comparison of EDIC between proton and photon therapy plans in patients with locoregionally advanced NSCLC treated at our institution.^18^, ^19^

## Methods

Data were collected and analyzed for 12 consecutive patients with locally or regionally advanced (clinically or biopsy-proven lymph node-positive, N1-3, and medically or surgically unresectable primary tumor) NSCLC treated between 2019 and 2021 who had physician-approved intensity-modulated radiotherapy (IMRT) photon therapy and intensity-modulated proton therapy (IMPT) treatment plans as part of concurrent conventionally-fractionated (chemo)radiotherapy. These patients typically had approved plans for both modalities as a result of either (1) anticipated prolonged delay to insurance approval for IMPT necessitating treatment start with IMRT or (2) prolonged cyclotron downtime necessitating temporary transition to IMRT.

Target delineation was similar for IMRT and IMPT plans. A 4DCT simulation scan was performed to allow for the creation of an internal gross tumor volume (iGTV). When available, positron emission tomography images were coregistered to facilitate tumor delineation. Per institutional practice, the iGTV was expanded by 5 mm to generate a clinical target volume (CTV), which was subsequently cropped from anatomical barriers to spread. IMPT planning was performed on Raystation (Raysearch Laboratories, Stockholm, Sweden) for delivery on a ProBeam proton system (Varian Medical Systems, Palo Alto, California). IMPT plans were robustly optimized on the CTV using 5% range uncertainty and 5 mm translational perturbations. Three to 5 beams were used for most plans. Single-field optimization planning technique was used to reduce the interplay effect from breathing motion. IMRT plans were generated after applying a 5 mm isotropic margin from the CTV to create a planning target volume. IMRT courses were prepared as volumetric modulated arc therapy plans on Eclipse software for delivery on a TrueBeam system (Varian Medical Systems, Palo Alto, California). Typically, 2 partial arcs were used in the IMRT plans.

Target planning and OAR risk optimization constraints were implemented on both IMRT and IMPT plans per institutional protocols (Supplementary Table 1). EDIC was not considered a unique OAR and was not constrained.

The necessary data to calculate EDIC using both the Jin and Ladbury models ((1), (2), respectively) were abstracted from the approved plans, including mean lung dose, mean heart dose (MHD), MBD, body volume, (ITD or body volume x MBD, depending on the model), and number of fractions.^12^, ^13^ These models differ in their final term, which represents a modeled dose to small vessels and capillaries throughout the body. Whereas Jin assumes a standard body volume of 63 kg (Eq. 1), Ladbury (Eq. 2) models this value using MBD, which is dependent upon the volume of the body scanned.^13^ Mean EDIC was compared between the 12 IMPT plans (in GyE) and the 12 IMRT plans (in Gy) with 2-sided paired *t* tests. The predicted 2-year OS (OS2) was also calculated as per the Jin model (Eq. 3).^12^

(1) $$\mathrm{EDIC}=0.12*\mathrm{MLD}+0.08*\mathrm{MHD}+\left[0.45+0.35*0.85*{\left(\frac{n}{45}\right)}^{\frac{1}{2}}\right]*\frac{\mathrm{ITD}}{62*10^{3}}$$

(2) $$\mathrm{EDIC}=0.12*\mathrm{MLD}+0.08*\mathrm{MHD}+\left[0.45+0.35*0.85*{\left(\frac{n}{45}\right)}^{\frac{1}{2}}\right]*\mathrm{MBD}$$

(3) $$2\mathrm{y OS}=0.74*\left[1-\frac{0.39}{1+{\left(\frac{4.5}{\mathrm{EDIC}}\right)}^{6}}\right]*\left[1-\frac{1}{1+{\left(\frac{9.9}{\mathrm{EDIC}}\right)}^{12}}\right]$$

## Results

**Figure 1:**
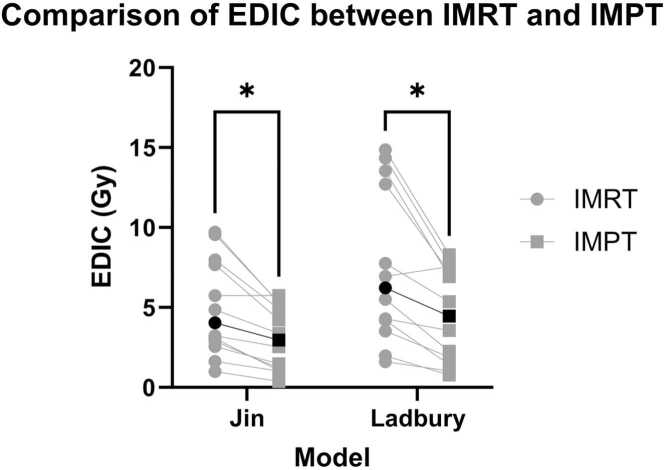
Comparison of EDIC between IMRT and IMPT between Jin and Ladbury models. *N* = 12 patients, each with physician-approved IMPT and IMRT plans. EDIC was calculated for each patient with both models and each circle (IMRT)/square (IMPT) pair represents a single patient. Lines between each paired circle and square show the change in EDIC between plans. Black points indicated median EDIC. *P ≤ .001 for Jin’s model and 0.002 for Ladbury’s model.

Our 12-patient cohort had a mean age of 69 years (range: 59-82 years), and most had N2 or greater nodal disease (67%). Most patients had right-sided (9/12) primary tumors (Table 1). The mean gross tumor volume was 143.3 cc (range: 0.9-536.7 cc, Table 2). One patient was prescribed 70 Gy(E) per RTOG1308, while the rest were prescribed 60 Gy(E). EDIC was found to be lower with IMPT than IMRT for 11 of 12 patients (92%). Mean EDIC across all 12 patients was significantly lower with IMPT than IMRT by both models (Figure 1**,** Table 3).

**Table 1:** Patient characteristics.

Pt	Gender	Age (Y)	Histology	Laterality	T- and N-classification (AJCC8)	Total dose (Gy/# fractions)
1	M	82	SCC	Left	T4N2	60/30
2	M	66	SCC	Left	T3N2	60/30
3	M	59	SCC	Right	T3N2	60/30
4	M	65	AC	Right	T2N2	60/30
5	M	58	SCC	Right	T2N3	60/30
6	M	71	AC	Right	T4N0	60/30
7	M	77	SCC	Right	T2N2	60/30
8	F	76	AC	Right	T3N1	60/30
9	F	76	AC	Right	T1N1	70/35
10	F	70	AC	Right	T2N1	60/30
11	M	60	SCC	Right	T3N3	60/30
12	F	70	AC	Left	T0N2	60/30

**Abbreviations:** AC, adenocarcinoma; F, female; M, male; SCC, squamous cell carcinoma.

**Table 2:** Doses to target volumes and organs at risk.

Pt	GTV (cc)	MHD (IMPT | IMRT, Gy)	MLD (IMPT | IMRT, Gy)	MBD (IMPT | IMRT, Gy)	ITDV (IMPT | IMRT, x1000 cc)
1	98.6	6.79 | 15.25	13.86 | 17.78	7.72 | 5.18	315.4 | 211.6
2	98.0	0.65 | 2.60	6.67 | 10.31	3.90 | 4.10	150.4 | 157.9
3	305.4	8.14 | 28.60	13.35 | 21.30	7.95 | 13.70	272.4 | 420.3
4	7.6	0.65 | 6.03	5.07 | 11.88	1.11 | 3.30	34.7| 103.2
5	536.7	6.13 | 22.82	16.57 | 25.80	8.38 | 14.34	249.3 | 426.7
6	0.9	0.00 | 0.50	7.89 | 10.79	1.36 | 3.15	46.7 | 108.3
7	379.5	0.73 | 1.45	9.67 | 13.09	5.97 | 8.77	192.3 | 282.5
8	152.7	6.02 | 22.86	18.49 | 26.98	6.49 | 11.02	136.7 | 232.1
9	0.8	2.62 | 7.60	4.44 | 9.80	2.11 | 5.21	38.8 | 95.7
10	12.7	1.00 | 3.79	4.95 | 5.84	0.50 | 0.88	31.7 | 55.8
11	126.6	4.89 | 24.80	16.74 | 21.88	6.54 | 12.89	223.9 | 301.0
12	1.3	0.48 | 0.60	1.37 | 4.30	0.82 | 2.04	15.2 | 37.7
Average		3.18 | 11.41	9.92 | 14.98	4.40 | 7.05	142.3 | 202.7
Median		1.81 | 6.82	8.78 | 12.49	4.94 | 5.20	143.6 | 184.8

**Abbreviations:** GTV, gross tumor volume; IMPT, intensity-modulated proton therapy; IMRT, intensity-modulated radiotherapy; ITDV, integral total dose volume; MBD, mean body dose; MHD, mean heart dose; MLD, mean lung dose.

**Table 3:** EDIC calculations.

Pt	Jin EDIC (IMPT | IMRT, Gy)	Excess EDIC (IMRT-IMPT), Gy	Ladbury EDIC (IMPT | IMRT, Gy)	Excess EDIC (IMRT-IMPT), Gy	2 yr OS (IMPT | IMRT, %)	Difference in 2-year OS with IMPT versus IMRT
1	5.74 | 5.74	+0	7.56 | 6.94	-0.62	50 | 50	+0%
2	2.54 | 3.22	+0.68	3.55 | 4.29	+0.74	73 | 71	+2%
3	5.31 | 9.56	+4.25	7.76 | 14.34	+6.58	53 | 21	+32%
4	1.05 | 3.06	+2.01	1.43 | 4.19	+2.76	74 | 71	+3%
5	5.27 | 9.71	+4.44	8.29 | 14.86	+6.57	53 | 25	+28%
6	1.47 | 2.55	+1.08	1.89 | 3.52	+1.63	74 | 73	+1%
7	3.38 | 4.85	+1.47	5.36 | 7.76	+2.4	70 | 56	+24%
8	4.23 | 7.67	+3.44	7.20 | 12.70	+5.5	62 | 44	+18%
9	1.19 | 2.89	+1.7	2.25 | 5.50	+3.25	74 | 72	+2%
10	1.03 | 1.63	+0.6	1.02 | 1.61	+0.59	74 | 74	+0%
11	4.91 | 7.98	+3.07	6.93 | 13.54	+6.61	56 | 43	+13%
12	0.37 | 0.99	+0.62	0.77 | 1.98	+1.21	74 | 74	+0%
Average	3.94 | 4.99	+1.05	4.50 | 7.60	+3.1	66 | 57	+9%
Median	2.96 | 4.04	+1.08	4.46 | 6.22	+1.76	71 | 63	+8%
	p < .001		p = .002		p = .03	

**Abbreviations:** EDIC, effective dose to immune cells; IMPT, intensity-modulated proton therapy; IMRT, intensity-modulated radiotherapy; OS, overall survival.

The modeled OS2 rate was found to be significantly lower with IMRT than IMPT using EDIC (median 63% vs 71%; *P* = .03). Just over half of the patients (7/12, 58%) had a modeled OS2 benefit with IMPT of less than 5%, while 5 (42%) had a modeled OS2 benefit exceeding 10%. The median modeled OS2 benefit with IMPT was +8% (Table 3).

## Discussion

In this dosimetric study comparing EDIC between IMPT and IMRT for patients with locally advanced NSCLC treated with definitive (chemo)radiation therapy, we found that IMPT offers a statistically significant reduction in EDIC as compared to IMRT in 11 of 12 consecutive patients.

Prior studies by Jin et al and Ladbury et al have identified an association between EDIC and survival outcomes among patients with locoregionally advanced NSCLC treated with definitive chemoradiotherapy.^20^ These studies raise the intriguing prospect of factoring the immune system into the treatment planning process as a distinct OAR and highlight a potential role for proton therapy. In our cohort, all 12 patients had an equal or reduced EDIC with IMPT versus IMRT per the Jin model. This differential held for all but one patient in the Ladbury model. The median decrease in EDIC with IMPT was 1.08 Gy and 1.76 Gy as calculated by the Jin and Ladbury models, respectively. Subtle differences in EDIC values between the 2 models are related to regarding assumptions of standard body volume (and ITDV) in the Jin model and of MBD in the Ladbury model, which is dependent upon the volume of body image in the planning CT study.

A few observations are worth noting from our case series. First, among the 3 addends in each EDIC equation, the MHD component appeared to feature the largest differences between IMPT and IMRT and therefore exerted the greatest influence on EDIC. Some of the largest differences in EDIC between IMRT and IMPT, however, were seen in patients with gross tumor volumes greater than 100 cc^3^ (eg, patients 3, 5, 7, 8, 11). The large reductions in EDIC with IMPT observed in these patients translated to clinically meaningful improvements in predicted OS2. These observations suggest that patients with bulkier or cardiac-adjacent disease may derive particular benefit from IMPT.

In support of this hypothesis, a recent retrospective study by Kim et al found that proton therapy for NSCLC was associated with less radiation-induced lymphopenia when compared to IMRT and that patients with severe radiation-induced lymphopenia experienced worse OS2.^21^ In a study of patients with locoregionally advanced esophageal cancer treated with neoadjuvant chemoradiotherapy, those receiving IMPT were found to have a lower risk of lymphopenia than those receiving IMRT.^22^ In addition, a recent analysis of patients with stage III NSCLC showed that IMPT can significantly reduce radiation dose to the heart and lungs as compared to IMRT, which both influence EDIC and modeled OS2.^23^ Additional studies are needed to more precisely model the association between EDIC, immune biomarkers, and survival outcomes to inform the selection of radiotherapy modality in UNSCLC.

There was one patient in our study for whom the EDIC was not lower with IMPT. While EDIC was numerically identical between IMRT and IMPT for this patient by the Jin model, it was higher with IMPT than IMRT by the Ladbury model. A closer analysis of this patient demonstrates that while IMPT entailed lower MHD and mean lung dose, there was an increase in MBD/ITDV. By comparing the 2 plans, we also noticed that there was a difference in the volume of the body that was scanned which may account for the observed difference in EDIC between the Ladbury model and the Jin model, as the latter uses a normalized body volume. This case illustrates the nonuniform effect of IMPT versus IMRT on EDIC and underscores the importance of radiotherapy modality selection for locally advanced NSCLC.

The Jin and Ladbury datasets predate the PACIFIC era: consolidative durvalumab is now the standard of care following chemoradiation in patients with locoregionally advanced NSCLC without progression after concurrent chemoradiation therapy.^1^, ^24^ Recent work at our institution has shown that in this population, higher EDIC correlated with worse OS, PFS, loco-regional control, and time to brain metastasis, perhaps with an even greater effect size as compared to studies that predate PACIFIC.^25^ This suggests that the immune-compartment-sparing advantage with IMPT we identify in this analysis may lead to even larger improvements in disease control and survival than previously modeled prior to the approval of durvalumab. A randomized study comparing IMRT versus IMPT among patients treated with durvalumab has recently completed accrual.^18^

Larger prospective randomized studies examining the effect of IMPT and IMRT on EDIC will be critical in generalizing our findings since our small sample size limits data evaluation and extrapolation to a larger population. Future prospective studies will also help elucidate clinical outcomes that may be suggested from our dosimetric calculations.

## Conclusion

IMPT offers a statistically significant reduction in EDIC and favorable predicted OS2 as compared to IMRT in patients with unresectable, locally advanced NSCLC. The impact of IMPT on OS2 appears variable among patients and may depend on disease volume and cardiac proximity. With EDIC emerging as a modifiable prognostic factor, our dosimetric study highlights a novel potential role for IMPT to improve outcomes in these patients.

## Author Contributions

**Jimmy S. Patel:** Methodology, Software, Formal Analysis, Investigation, Writing – Original Draft, Writing – Review & Editing. **Neal S. McCall:** Methodology, Formal Analysis, Investigation, Writing – Original Draft, Writing – Review & Editing. **Matthew Thomas:** Methodology, Software, Formal Analysis, Investigation, Writing – Review & Editing. **Jun Zhou:** Methodology, Software, Formal Analysis, Investigation, Writing – Review & Editing. **Kristin A. Higgins:** Resources, Writing – Review & Editing. **Jeffrey D. Bradley:** Resources, Writing – Review & Editing. **Sibo Tian:** Resources, Writing – Review & Editing. **Mark W. McDonald:** Resources, Writing – Review & Editing. **Aparna H. Kesarwala:** Conceptualization, Methodology, Formal Analysis, Writing – Original Draft, Writing – Review & Editing, Supervision. **William A. Stokes:** Conceptualization, Methodology, Formal Analysis, Writing – Original Draft, Writing – Review & Editing, Supervision.

## Declaration of Conflicts of Interest

JSP is supported by NIH T32CA275777. NSM, MT, JZ, and WAS have no disclosures. KAH reports financial support provided by AstraZeneca Pharmaceuticals LP, Regeneron Pharmaceuticals Inc, Jazz Pharmaceuticals Inc, Janssen Pharmaceuticals Inc, and Picture Health. JDB reports a relationship with Mevion Medical Systems that includes: consulting or advisory; Varian Medical Systems Inc that includes: consulting or advisory, funding, and grants; AstraZeneca Pharmaceuticals LP that includes: consulting or advisory; Genentech Inc that includes: consulting or advisory. ST reports a relationship with Genentech that includes: consulting or advisory; Varian Medical Systems Inc that includes: funding grants; Merck & Co Inc that includes: consulting or advisory. MWM reports a relationship with the National Association for Proton Therapy that includes: board membership. AHK reports a relationship with the National Institutes of Health that includes: funding.

## Data availability

The data that support the findings of this study are available from the corresponding authors upon reasonable request.
