# Supplementary material for: Immune System Dose With Proton Versus Photon Radiotherapy for Treatment of Locally Advanced NSCLC
Source: Int J Part Ther. 2024 Apr 23;12:100016. doi: 10.1016/j.ijpt.2024.100016 (PMC11145531; doi:10.1016/j.ijpt.2024.100016)
Supplement: Supplementary file 1 — Supplementary material [file mmc1.docx]

**Supplemental Table 1 – Treatment Planning OAR constraints**

| OAR | Treatment Planning Constraints | Priority** |
| --- | --- | --- |
| Brachial Plexus | V_66GyE_ = 0% | 2 |
| Esophagus | Mean dose < 34 GyE  V_63GyE_ = 0% | 2  2 |
| Heart | Mean dose < 20 GyE  V_30GyE_ < 50%  V_45GyE_ < 30%  V_63GyE_ = 0% | 2  2  2  2 |
| Lungs | Mean dose < 20GyE  V_5GyE_ < 60%  V_20GyE_ < 35% | 1  2  1 |
| Spinal Cord | V_45GyE_ = 0% | 1 |
| Trachea | V_63GyE_ = 0% | 2 |

* Percentages refer to total volume of contoured OAR

** 1 = Prioritize over other OARs and may need to compromise target coverage. 2 = prioritize without compromise to target coverage.
